# Clinical features and treatment outcome in newly diagnosed Chinese patients with multiple myeloma: results of a multicenter analysis

**DOI:** 10.1038/bcj.2014.55

**Published:** 2014-08-15

**Authors:** J Lu, J Lu, W Chen, Y Huo, X Huang, J Hou, the Chinese Medical Doctor Association Hematology Branch

**Affiliations:** 1 Department of Hematology, People's Hospital, Beijing University, Beijing, China; 2 Department of Hematology, The Myeloma & Lymphoma Center, Changzheng Hospital, The Second Military Medical University, Shanghai, China; 3 Department of Hematology, Beijing Chaoyang Hospital, Capital Medical University, Beijing, China; 4 Beijing University Clinical Research Institute, Beijing, China

## Abstract

The aim of this study was to understand the clinical features and treatment outcome of Chinese patients with multiple myeloma (MM). This retrospective study enrolled 940 newly diagnosed inpatients (median age, 59 years; immunoglobulin (Ig)D isotype, 6.5%) with complete follow-up data at three centers. In all, 85.8% of patients were of Durie-Salmon stage III and 48.3% were of International Staging System (ISS) stage III at diagnosis. Also, 9.6% of patients had extramedullary plasmacytoma. Compared with IgG, IgD-type patients were diagnosed at a younger age, and more patients were of ISS stage III, with hypercalcemia, elevated levels of lactate dehydrogenase, hyperuricemia, renal dysfunction and 1q21 amplification (*P*=0.03). The overall survival (OS) benefit was more prominent in IgG than in IgD when patients received bortezomib; however, they showed no significant difference when they received older therapies such as melphalan combined with prednisone or vincristine combined with adriamycin and dexamethasone. Fluorescence *in situ* hybridization (FISH) results showed that 17.6% had 17p13 deletion. Conventional cytogenetics revealed that 13.3% were hypodiploid and those cases had the worst survival, but hyperdiploid cases (9.3%) did not show any survival benefit compared with those with a normal karyotype (77.4%). Median OS and progression-free survival for all patients were 54 and 26 months, respectively. Significant factors for survival by multivariate analysis were gender, ISS stage, number of FISH abnormalities and extramedullary disease. MM in mainland China presents with different features, with patients being of younger age and having higher risk and more survival benefit in IgG patients receiving bortezomib.

## Introduction

Multiple myeloma (MM), a cancer derived from plasma cells, ranks second among hematological malignancies in many countries. In 2011, 20 520 people were diagnosed with MM in the USA, and 10 610 people died as a result of it.^1^ The exact number of cases in China is not known. If the Japanese incidence of multiple myeloma is applied to China, there would be an estimated 27 800 new cases (1.39 billion × 2/100 000) each year and a total of 200 000 cases in China. With the acceleration of the aging process in China, it is predicted that MM, with a rapid growth in incidence, will become one of the more significant diseases that affect people's health in the country.

The occurrence, type and outcome of the cancer is closely related to factors such as race, environment and so on. A higher incidence of MM has been reported in African Americans than in whites,^1^ and in the SEER analysis the Asian population had the best outcome compared with other races.^2^ However, it has been reported that immigrants from Asia living in the USA have an increased incidence of MM compared with those living in Asia.^3^ The reason for this is that both race and environmental factors are important determinants in the etiology of MM. Also, markers for prognostic evaluation can be different because of differences in race^4^ and diet.^5^ At present, no large, multicenter epidemiological study for Chinese MM patients is available, with some recognized prognostic factors still unknown, such as International Staging System (ISS), cytogenetic results, plasma cell immunological markers, heavy chain types and so on. As a result of new drugs such as bortezomib and thalidomide, the complete remission rate and long-term survival rate have improved over the past 10 years, with the 5-year (between 1994 and 2006) survival rate increasing from 24 to 34%.^6^ However, the effects of these drugs in Chinese patients, the remission duration and retreatment after relapse still need to be understood.

Therefore, we undertook a multicenter retrospective study to understand these aspects better and develop a more targeted plan for the future. This was a chart review study across three centers. Over 70% of patients at these three centers were from across the country, with all centers being affiliated hospitals of medical universities. Patients initially suspected of having MM in local hospitals are subsequently referred to these study hospitals.

## Patients and methods

### Study population and definition

The study continuously enrolled newly diagnosed inpatients at the three centers from 1 January 2008 to December 2011 with complete follow-up data. The hospitals were Peking University People's Hospital, Shanghai Changzheng Hospital and Beijing Chaoyang Hospital. This study was retrospective and did not intervene in the patients' treatment and all data were unmasked. Therefore, informed consent was not sought. The study was approved by the Clinical Research Ethics Approval Committee of Peking University People's Hospital.

The cases were those with a diagnosis of MM confirmed by a pathologist. The information consisted of 78 data points, including age, gender, subtype, initial symptoms, especially the incidence of peripheral neuropathy, Durie–Salmon (DS) and ISS staging, bone destruction number, whether complicated with other plasma disease such as POEMS syndrome and amyloidosis, whether combined with extramedullary plasmacytoma/disease (EMD) and its location, the results of chromosome G-banding and interphase fluorescence *in situ* hybridization (FISH) at diagnosis, such as whether RB1 deletion, the amplification of 1q21, IgH recombination, p53 deletion and D13S319 deletion are positive, and their values. Cell sorting was performed by miniMACS (Miltenyi Biotec, Bergisch Gladbach, Germany) as per the manufacturer's instructions. Other parameters including white blood cell count, platelet count, bone marrow plasma cell number, peripheral blood plasma cell percentage, quantitative and subtype analysis of M protein in serum or urine, erythrocyte sedimentation rate, lactate dehydrogenase, uric acid, creatinine, C-reactive protein, β2-MG, calcium and albumin were also included. Information such as diagnosis time, initial treatment, the treatment of different lines and the efficacy and the results of immunophenotyping (CD38, CD138, CD56, CXCR4, CD19, CD20, CD117, CD9, CD45, CD22, CD28 and CD200) were also collected. To optimally detect antigen expression, we set plasma cells according to CD138, CD38 and CD45. The initial analysis gate was devised using CD38 versus CD138 expression, and the second one using CD38 brightly positive versus CD45-positive and -negative cells. Cases were considered positive if the percentage of plasma cells showing positive expression for a given antigen was >20%. Overall survival (OS) and progression-free survival (PFS) were calculated. The definition of EMD was one of the following: pleural effusion with clonal plasma cells in pleural fluid, with clonal plasma cells in ascitic fluid or with clonal plasma cells in cerebrospinal fluid; biopsy from organ or tissue demonstrating plasmacytoma; computed tomography- or positron emission tomography–computed tomography-confirmed plasmacytoma (plasmacytoma that extended beyond a bone was not included). The diagnosis of peripheral neuropathy was made on the basis of symptoms such as pain, numbness and hypoaesthesia, some of which were confirmed by electromyography. The rationale for comparing IgG and IgD myeloma was to compare the baseline characteristics, including all the information shown above, and the treatment outcome. The International Myeloma Working Group criteria were used to define response rate and event end point. PFS was the duration from the start of treatment to disease progression or death, whichever came first. For OS, the time interval was measured from the date of diagnosis to the date of death or last follow-up. Death from all causes was included.

### Statistical analysis

Survival time was measured until 30 March 2013. The Chi-square test, *t*-test and Fisher's exact test were used in the comparison of demographic characteristics. We constructed a Cox proportional hazards model to evaluate the association between patient characteristics and OS. Hazard ratios and 95% confidence intervals were generated, with hazard ratio <1.0 indicating survival benefit (or reduced mortality). Data analysis was performed using SPSS version 18.0 (Chicago, IL, USA), and statistical significance was set at *P*<0.05 (two-sided).

## Results

### Patient characteristics and laboratory findings

Data from a total of 1153 cases were collected, with 940 cases remaining after the exclusion of incomplete cases. An overall 14.4% had received an autologous transplant. The patient characteristics are summarized in Table 1. The median age was 59 years (23–88 years). There were 570 male (60.6%) and 370 (39.4%) female patients, with a male to female ratio of 1.54:1, the mean age of males being 59.52 years and that of females being 59.09 years; there were no significant differences between genders (*P*=0.87).

As for the MM subtype, IgG type was 44.3%, light chain type was 27.5% and IgD type was 6.5%. The proportion of patients at DS stage III and ISS stage III was 85.8% and 48.3%, respectively. Of the patients, 11.7% had peripheral neuropathy at diagnosis. About 9.6% (90/940) of patients had extramedullary plasmacytoma at initial diagnosis: 25 cases in soft tissue (upper arm, ectopectoralis, gum, retrobulbar, hips, chest wall), 23 cases in the vertebral canal, 20 cases in pleural effusion and ascitic fluid, 17 cases in organs (lung 8/liver 7/spleen 1/testicle 1), 1 case in blood and 4 cases in the central nervous system.

### IgD characteristics

Among the 61 cases of IgD-type MM, only 3 cases (5%) were IgD kappa and 58 cases (95%) were IgD lambda. When compared with IgG (the most common type), the IgD type was seen in younger patients, and more frequently in patients at ISS stage III (80.3% vs 44.5%) and in those with 1q21 amplification (*P*=0.03). In addition, compared with IgG, the IgD patients more commonly had hypercalcemia, elevated lactate dehydrogenase and hyperuricemia (*P*<0.001), and 45% of patients had renal dysfunction at diagnosis. However, median values of white blood cells, hemoglobin and platelets were lower than in patients with the IgG isotype at diagnosis (*P*<0.001). There were no significant differences in gender, DS stage, conventional cytogenetic abnormalities (G-banding showing hyperdiploid or hypodiploid) and percentage of EMD, nor in the RB1 deletion, IgH rearrangement, p53 deletion and 13q14.3 deletion detected by FISH, between IgD and IgG types. The results are shown in Table 2.

When compared with IgA, the IgD isotype was seen in younger patients (*P*<0.001), was more common in patients with renal dysfunction (*P*<0.001) and had more patients at ISS stage III (*P*<0.001). When compared with light chain isotype, the IgD isotype was more common with 1q21 amplification (*P*=0.01) and had more patients at ISS stage III (*P*<0.001) (the results are shown in Supplementary Tables S1 and S2).

**Figure 1:**
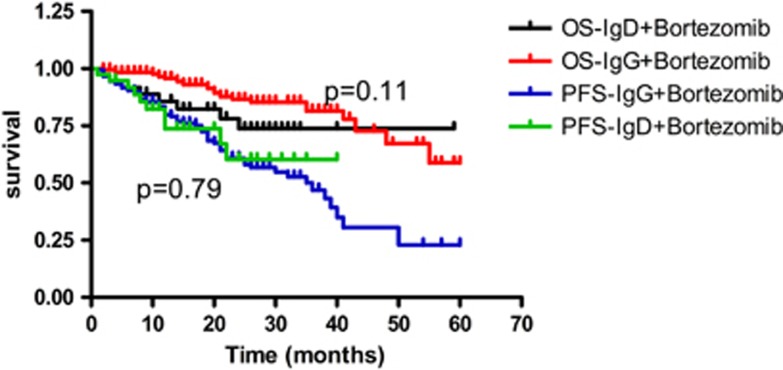
OS and PFS in IgG and IgD patients who received bortezomib.

**Figure 2:**
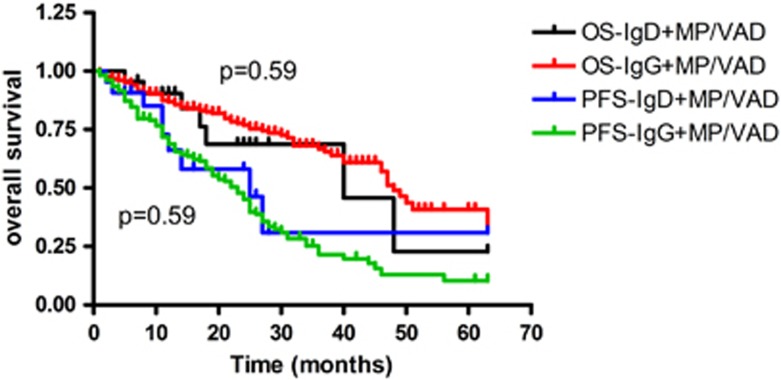
OS and PFS in IgG and IgD patients who received MP and VAD.

**Figure 3:**
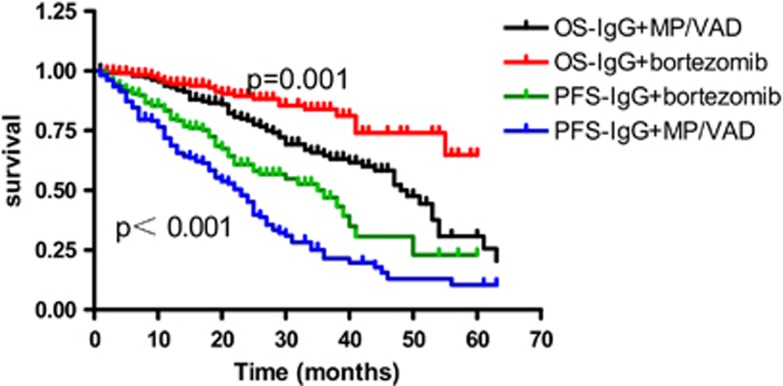
OS and PFS in IgG patients who received bortezomib and MP/VAD.

For patients who received bortezomib treatment, median OS was not reached. The mean OS for IgG and IgD was 50 and 47 months, respectively (*P*=0.11; Figure 1, when the Breslow method was used to analyze the survival difference, *P*=0.03), and the median PFS for both was 36 months (*P*=0.79). When the patients received MP (melphalan combined with prednisone) or VAD (vincristine combined with adriamycin and dexamethasone) treatment regimens, the median OS for IgG and IgD was 48 and 40 months, respectively (*P*=0.59; Figure 2). The PFS was 23 and 25 months, respectively (*P*=0.59; Figure 2). Whether or not IgD patients received bortezomib treatment, they showed no significant difference in OS (*P*=0.55) and PFS (*P*=0.30). IgG patients who received bortezomib treatment achieved survival benefit, not only for PFS but also for OS (*P*<0.001 and *P*<0.001, respectively; Figure 3).

### Cytogenetic results

**Figure 4:**
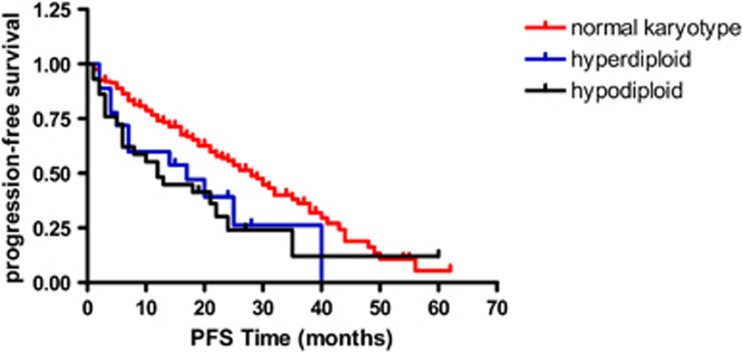
PFS for patients with hyperdiploid, hypodiploid and normal karyotypes.

**Figure 5:**
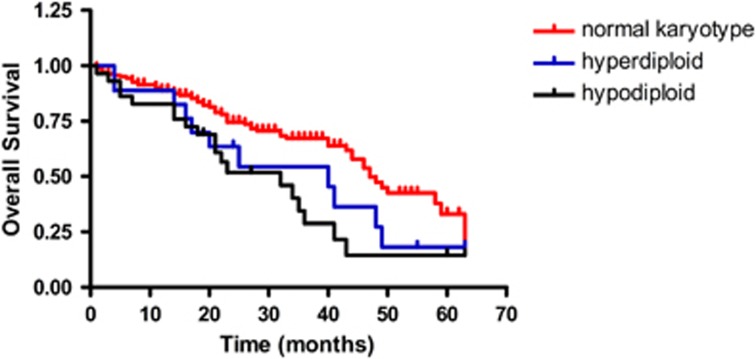
OS for patients with hyperdiploid, hypodiploid and normal karyotypes.

There were 241 cases with routine cytogenetic results: 9.3% were hyperdiploid, 13.3% were hypodiploid and 77.4% were of normal karyotype. Hypodiploid had the worst survival, but hyperdiploid did not show greater survival benefit than the normal karyotype (see Figures 4 and 5). A total of 442 cases had FISH results: FISH results showed RB1 deletion in 39.9% (71/178), 1q21 amplification in 42% (168/400), IgH rearrangement in 55.7% (226/406), P53 deletion in 17.6% (78/442) and 13q14 deletion in 39.66% (161/406) of cases. Among them, 41.5% of cases were sorted by CD138^+^ magnetic beads. When comparing the sorted and non-sorted cases, the frequency was as follows: the RB1 and 13q14.3 deletion was not done in the sorted samples, the 1q21 was 43.3 and 40.3% (*P*=0.44), the IgH rearrangement was 59 and 51.4% (*P*=0.17) and the p53 deletion was 23.1% in the sorted and 9.6% in the non-sorted cases (*P*<0.001). p53 deletion had the worst survival (see Supplementary Figure S1): the median PFS was 23 and 29 months (*P*<0.001), respectively, in those with or without p53 deletion.

### Immunophenotypes detected with flow cytometry

CD38^+^ 99.7% (323/324), CD138^+^ 99.1% (326/329), CD56^+^ 64.5% (195/302), CXCR4^+^ 34.7% (78/225), CD20^+^ 22.7% (61/268), CD117^+^ 41% (111/271), CD9^+^ 62.5% (150/240), CD45^+^ 15.5% (36/232), CD22^+^ 6.90% (2/29), CD28^+^ 20.3% (35/172), CD200^+^ 58% (65/112).

### Treatment outcome

A diverse range of first-line treatments was used because patients were treated differently in the centers over the 4 years. This was partially affected by the patient's financial standing because bortezomib is not covered by insurance in China and thalidomide was off-label used in MM. The median line of treatment was 3 (range 1–6). Among them, only 14.4% of patients had autologous stem cell transplantation (ASCT) after induction therapy, all of them younger than 65 years. ASCT was performed routinely after 3–4 cycles of induction therapy as part of a total therapy protocol. The median time from diagnosis to ASCT was 8 months (5–12 months). The conditioning regimen was Mel 200 mg/m^2^ in the majority of patients and CBV (cyclophosphamide combined with BCNU and etopside) in some patients. There were 644 cases younger than 65 years among them and 135 cases received ASCT. In patients younger than 65 years, the median OS was 59.17 months among those who received ASCT and 50.27 months among those who did not (*P*<0.001) and the median PFS was 38 and 27 months (*P*<0.001), respectively; for patients older than 65 years, the median OS was 46.07 months and PFS was 21 months. When compared with patients younger than 65 years, these older patients had inferior survival, whether or not they received transplantation (as shown in Supplementary Figure S4–9). A bortezomib-containing regimen was used in 468 cases (49.8%); bortezomib combined with adriamycin and dexamethasone, bortezomib combined with dexamethasone and bortezomib combined with dexamethasone and thalidomide were the most commonly used ones. A thalidomide-containing regimen was used in 272 cases (28.9%); thalidomide combined with dexamethasone and thalidomide combined with adriamycin and dexamethasone were the most commonly used ones. VAD and MP were used in 152 cases (16.2%). Forty-eight cases (5.1%) abandoned therapy or died before receiveing MM therapy. After induction or ASCT, the patients routinely received thalidomide or interferon-γ for maintenance therapy. The median OS for all patients was 54 months and the median PFS was 26 months. Patients who received bortezomib as first-line treatment had better PFS and OS compared with those who received older therapies such as MP and VAD. The median PFS for patients who received bortezomib as first-line treatment was 35 months, and 20 months for patients who received MP or VAD as first-line therapy (*P*<0.001). The median OS for patients who received the bortezomib-containing regimen was 58 months and that for patients who received MP or VAD was 49 months (*P*=0.04). An overall 54.4% young patients received the bortezomib-containing regimen compared with 38.8% older patients (*P*<0.001). The PFS benefit from bortezomib could be seen only in younger patients but not in older ones; the median PFS was 38 and 25 months (*P*<0.001), respectively, in younger patients who received bortezomib and those who did not. For patients older than 65 years, the median PFS was 22 and 21 months, respectively (*P*=0.12).

### Survival

We analyzed gender, age, DS and ISS stage, renal dysfunction, peripheral neuropathy, the number of bone lesions, other accompanying plasma diseases, extramedullary disease, the number of FISH abnormalities, whether the patient had received or not received autologous transplantation, and the treatment regimen using univariate and multivariate analysis. The results showed that in univariate analysis the significant factors were gender (*P*<0.001), age (*P*<0.001), DS stage (DS stage III vs II and I, *P*<0.001) and ISS stage (ISS stage III vs II and I, *P*<0.001), extramedullary disease (*P*<0.001) and the treatment, including whether the patient had received autologous transplantation (*P*<0.001) and the regimen (bortezomib or not, *P*<0.001). The number of FISH abnormalities (1 vs 2 or more, *P*=0.11), the number of bone lesions (bone lesion 3 or more vs 2 and 1, *P*=0.21) and whether the patient had renal dysfunction (*P*=0.41) did not show any prognostic value on survival. The median OS was 44 months for ISS stage 3, and was not reached for ISS stage I and stage II (*P*<0.001) (see Supplementary Figure S2). The median OS was 49.2 months for DS stage III, and was not reached for DS stage I and stage II (*P*<0.001) (see Supplementary Figure S3). With multivariate analysis, the significant factors were gender (*P*=0.01), age (*P*<0.001), ISS stage (*P*=0.00), extramedullary disease (*P*=0.00), the regimen (bortezomib or not, *P*=0.03) and ASCT (*P*=0.03) (see Table 3).

## Discussion

MM is a plasma cell malignant disease with a high degree of heterogeneity, not only in its clinical manifestation but also in survival. The reason for this is the difference in its biological characteristics among patients. MM is closely correlated with ethnicity,^2,4^ environment and race; the same race living in a different country will have different clinical spectrums. For example, in the USA, a higher incidence of MM has been reported in African Americans than in whites, and the Asian population has a better outcome than do other races. Immigrants from Asia living in the USA have an increased incidence of MM compared with those living in Asia. Different races have different metabolic systems, such as more 2C19 slows down metabolic type in the Asian population, and the drugs may have different pharmacokinetics/pharmacodynamics in different races. As a result of the discovery of new drugs such as bortezomib and thalidomide, the complete remission rate and long-term survival rate have improved worldwide over the past 10 years. However, the effect of these drugs in Chinese patients is less understood and the remission duration is still unknown. Thus, we need to know the spectrum of MM disease and the effect of treatments in China. To our knowledge, there has not been a large multicenter epidemiological study of Chinese MM patients until now. Only local data are available,^7,8^ and therefore we organized this study to resolve those unanswered questions.

In this retrospective study, we analyzed 940 newly diagnosed MM cases in 4 years. The median follow-up time was 21 months (1–63 months).

Previous populations have shown variations in disease incidence and survival among patients from different ethnicities and geographical backgrounds. In the SEER analysis the median age was 69 years, but in Chinese patients the median age was only 59 years, much younger than patients in the USA. The SEER analysis showed that female Asian patients were of a lower age than male Asian patients, which was different from what has been observed in other ethnicities. In our retrospective analysis we found the same phenomenon: the mean age of males was 59.52 years and that of females was 59.09 years, but there was no significant difference between genders (*P*=0.87).

The largest case study available is that of Greipp *et al.*^9^ published in 2005. Compared with that study, the Chinese had more patients in late-stage DS III (85.8% vs 66%) and ISS III (48.3% vs 39%), more IgD patients (6.5% vs 1%), more light chain patients (27.5% vs 11%) and fewer patients with IgG type (44.3% vs 60%). The proportion of p53 deletion patients was higher than previously reported (17.6% vs 10%).^10,11^ When comparing FISH results between sorted and non-sorted samples, the results show that sorted samples have a higher proportion of p53 deletion (23.1% vs 9.6%).

Bone destruction, defined as the number of bones affected, was 0 for 33.1%, 1–2 for 22.1% and ⩾3 for 44.8% of our group. The three hospitals used plain X-ray as the main screening method. The screening method may not have been completely the same across centers and the number of cases without bone damage may have been overestimated. An overall 9.6% of patients had complicated EMD, higher than that of western relapse patients, which has been reported at only 7%,^12^ but in accordance with other reports from China.^13^ The patients who had EMD had the worse survival. Not only univariate but also multivariate analyses showed that EMD was an independent prognostic factor for survival. Patients who had accompanying peripheral neuropathy were 11.7%, but the incidence could be higher because a description or inquiry about peripheral neuropathy may not have always been made in this retrospective analysis. The possible reason for such a high incidence of peripheral neuropathy could be that the retrospective analysis had some defects, and the delay in the diagnosis. Because of the delay in diagnosis, 68% of patients had bone pain and 44.8% had a combined pathological fracture, making it impossible to distinguish.

IgD MM is rare, comprising only 1–2% of all myeloma cases in many countries. However, in China, we saw 6.5% IgD MM cases. The IgD MM patients in China were of younger age, and the median age was much younger than reported (61 vs 55 years).^14^ Patients were almost totally restricted to lambda light chain (95%), and more often had hypercalcemia, elevated lactate dehydrogenase and hyperuricemia. There were more patients at ISS stage III and 45% of patients had renal dysfunction at diagnosis. With regard to cytogenetics, there were more IgD isotype patients with 1q21 amplification. For patients who received bortezomib treatment, the survival benefit was more obvious in IgG than in IgD patients. Several studies have suggested that IgD may be sensitive to bortezomib,^15,16^ but this was not shown in our study. In our analysis, bortezomib did not bring more benefit compared with older therapies, but because of limited cases there may be some bias (only 27 patients with IgD received bortezomib treatment), and this finding needs further validation. IgD MM had a poorer prognosis than IgG, IgA and the light chain isotype, and our analysis also showed that IgD had the worst survival. Many trials have shown that cytogenetics have an important role in the prognosis of MM. Our results showed that when using conventional cytogenetic techniques only 22.6% of patients had positive results. Among them, hyperdiploid comprised 9.3% and hypodiploid comprised 13.3%, and the majority had the normal karyotype. Hyperdiploid patients did not have a survival benefit over those with a normal karyotype. Considering the slow proliferation feature of the plasma cell, we believe that patients who had positive results in G-banding, either hyperdiploid or hypodiploid, had high-grade proliferation and therefore would have worse survival than patients of the normal karyotype.

In all, 49.8% of those who received bortezomib as first-line therapy had better OS compared with those who did not receive bortezomib treatment, partially because more younger patients received a bortezomib-containing regimen. When we analyzed by age, the PFS benefit from bortezomib could be seen only in younger patients but not in older ones; the median PFS was 38 and 25 months (*P*=0.00), respectively, for younger patients who received bortezomib compared with those who did not. For patients older than 65 years, the median PFS was 22 and 21 months (*P*=0.12). There was no significant difference in OS between younger and older patients. There were no significant differences between MP or VAD when used as first-line treatment. The prognosis of patients was related to age, the treatment option (bortezomib or not) and ISS stage, but was not related to the subtype of the disease and the plasma cell burden. In our analysis the FISH results were confirmed as independent prognostic factors in multivariate analysis.

There were some limitations in this study. First, this was a retrospective study; some initial symptoms may have been underevaluated, such as peripheral neuropathy and bone destruction, and the true figures may be higher than reported. Second, the treatment was not uniform, and therefore survival may have been different when analyzed under the same regimen. Third, for economic reasons, the patients may have stopped treatment before the maximum response had been reached, especially patients receiving bortezomib, and survival may have been affected by this.

In conclusion, this study, to date, is the largest epidemiological study conducted in China at three major myeloma centers and shows that MM in mainland China has a different pattern, affects younger patients, is a more high-risk disease, has more patients at DS stage III and ISS stage III of disease and has shorter survival, and that bortezomib can lead to better PFS.

## Figures and Tables

**Table 1:** Baseline patient characteristics

*Characteristics (*n*=940)*	n *(%)*
*Age (years)*	
Median	59
Range	23–88
	
*Gender*	
Male	570 (61.0)
Female	370 (39.0)
	
*Type*	
IgA	169 (18.0)
IgG	416 (44.3)
IgD	61 (6.5)
Non-secretory	27 (2.9)
Light chain	259 (27.5)
Other type	8 (0.8)
	
*Bone destruction*	
0–1	307 (33.1)
2	205 (22.1)
⩾3	416 (44.8)
	
*DS staging*	
IA	26 (2.8)
IB	2 (0.2)
IIA	94 (10.1)
IIB	10 (1.1)
IIIA	584 (62.8)
IIIB	214 (23.0)
Missing	1.10%
	
*ISS staging*	
I	181 (19.9)
II	288 (31.7)
III	439 (48.3)
Missing	3.40%
	
Extramedullary plasmacytoma/disease	90 (9.6)

Abbreviations: DS, Durie–Salmon; Ig, immunoglobulin; ISS, International Staging System.

**Table 2:** Baseline characteristics between IgD and IgG isotypes

	*IgG isotype*	*IgD isotype*	P
*n*	416	61	
Lambda (%)	45.9	95	0
Gender (male, %)	59.4	62.3	0.66
Age (median (range)) (years)	61 (27–88)	55 (38–75)	0
DS I/II/III (%)	5.1/13.2/81.7	0/8.2/91.8	0.09
Renal dysfunction (%)	15.9	44.3	0
ISS I/II/III (%)	18.2/37.3/44.5	4.9/14.8/80.3	0
Extramedullary disease (%)	13.6	9.8	0.43
WBC (median (range)) ( × 10^9^/mm^3^)	4.9 (0.3–29.1)	4.9 (0.15–15.9)	0
Hb (median (range)) (g/l)	90.4 (12–161)	76 (10–127)	0
Plt (median (range)) ( × 10^9^/mm^3^)	166 (11–620)	119 (17–442)	0
LDH (median (range)) (IU/l)	164 (46–1065)	186 (90–1622)	0
Uric acid (median (range)) (IU/l)	369 (5–1000)	447 (188–911)	0
Creatinine (median (range)) (μmol/l)	80 (0–1115)	160 (33–890)	0
CRP (median (range)) (mg/l)	3.08 (0–170)	2.89 (0.1–76.2)	0
Calcium (median (range)) (mmol/l)	2.2 (0–198)	2.23 (1.54–3.91)	0
Alb (median (range)) (g/l)	32 (0–51)	37.2 (14.5–49)	0
Percentage of plasma cells in bone marrow (%, range)	25 (0–97.5)	34.5 (5–94)	0.23
Conventional cytogenetic abnormalities (%)	13.9	14.8	0.87
RB1 deletion in FISH (%)	40.2	50	0.79
1q21 amplification in FISH (%)	38.9	60	0.03
IgH rearrangement in FISH (%)	52.1	56.7	0.64
p53 deletion in FISH (%)	15.2	24.2	0.2
13q14 deletion in FISH (%)	38.3	34.3	0.84

Abbreviations: Alb, albumin; CRP, C-reactive protein; DS, Durie–Salmon; FISH, fluorescence *in situ* hybridization; Hb, hemoglobin; Ig, immunoglobulin; ISS, International Staging System; LDH, lactate dehydrogenase; Plt, platelet; WBC, white blood cell.

**Table 3:** Multivariate analysis of overall survival

	*HR*	*95% CI*	P*-value*
Gender	1.57	1.14–2.15	0.01
Age	1.03	1.02–1.05	0
Bortezomib or not	0.73	0.54–0.97	0.03
DS	0.57	0.33–0.99	0.05
ISS	0.48	0.35–0.65	0
EMD	0.49	0.35–0.68	0.01
ASCT or not	1.98	1.07–3.64	0.03

Abbreviations: ASCT, autologous stem cell transplantation; CI, confidence interval; DS, Durie–Salmon; EMD, extramedullary plasmacytoma/disease; HR, hazard ratio; ISS, International Staging System.
