# Supplementary figures and images for: Clinical features and treatment outcome in newly diagnosed Chinese patients with multiple myeloma: results of a multicenter analysis
Source: Blood Cancer J. 2014 Aug 15;4(8):e239–. doi: 10.1038/bcj.2014.55 (PMC4219472; doi:10.1038/bcj.2014.55)

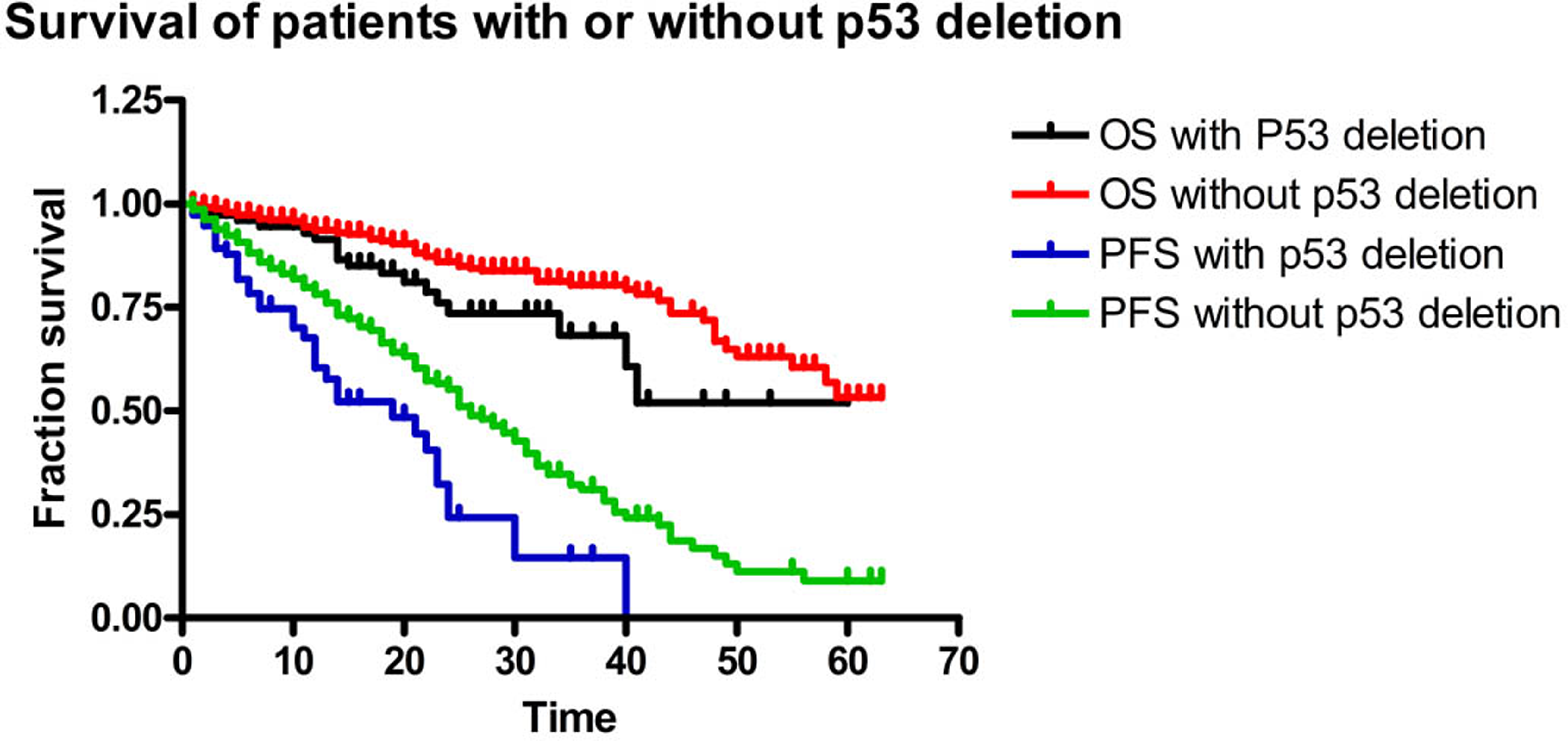

Supplement: Supplementary Figure 1 [file bcj201455x1.tif]

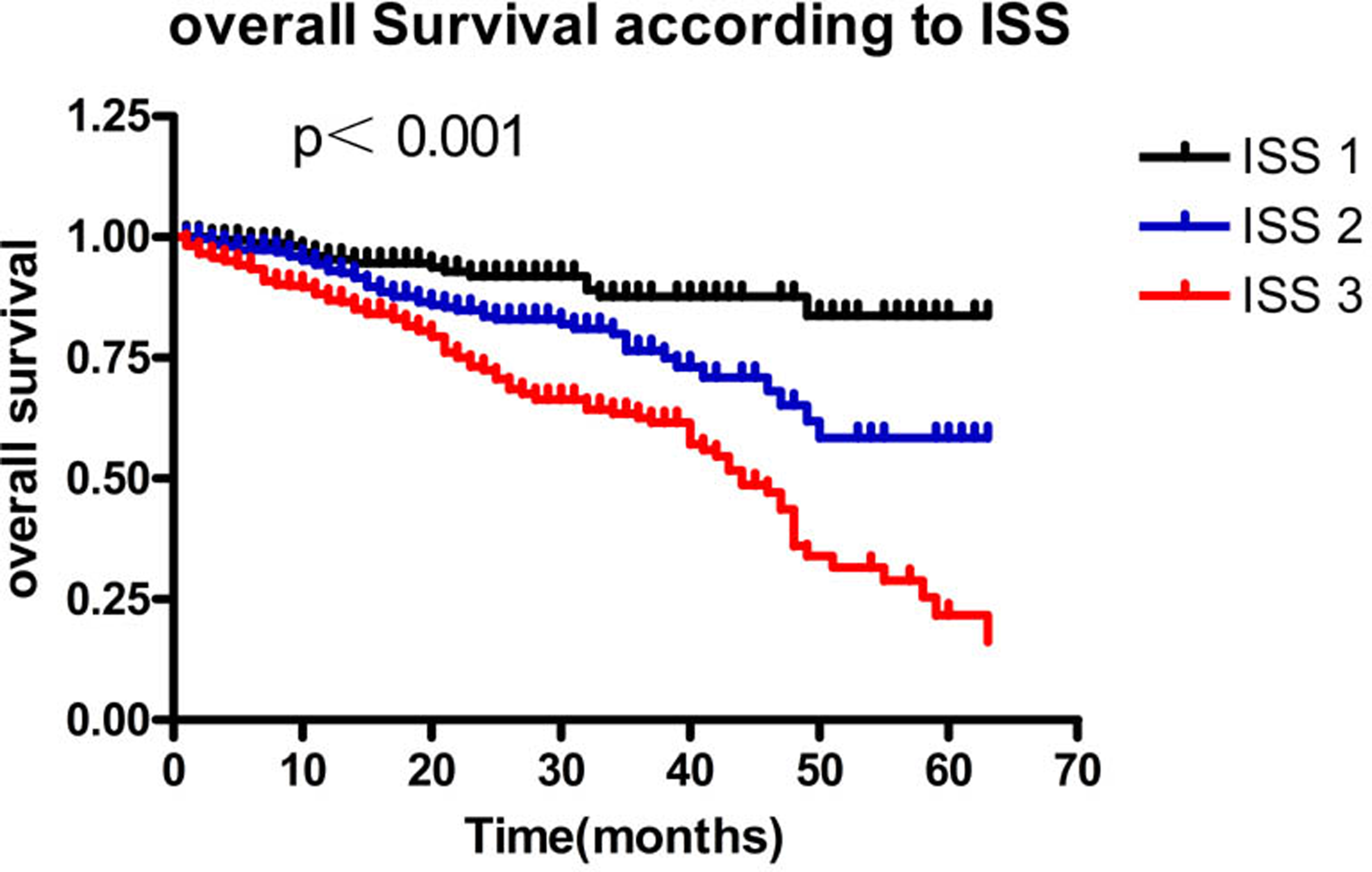

Supplement: Supplementary Figure 2 [file bcj201455x2.tif]

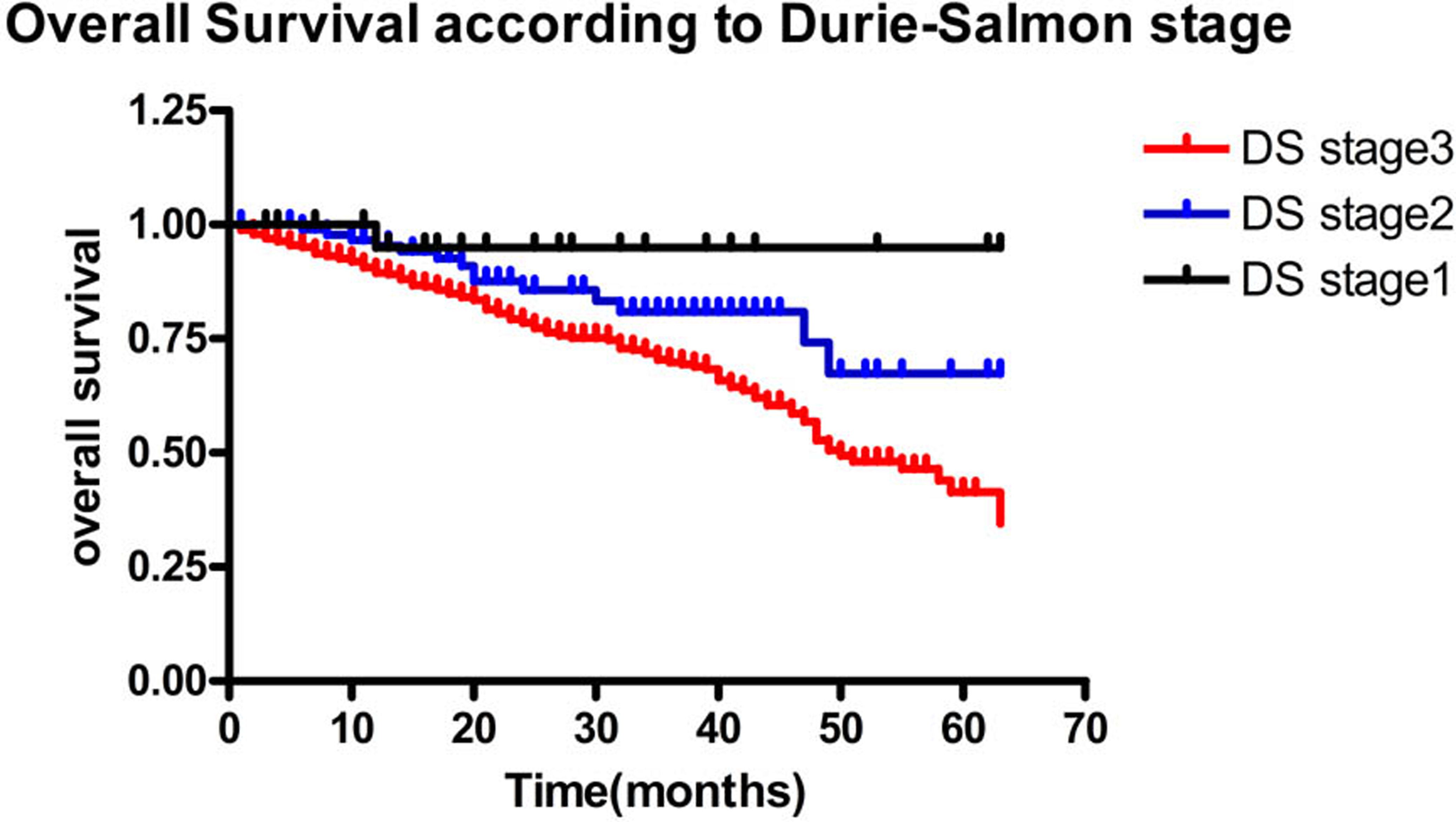

Supplement: Supplementary Figure 3 [file bcj201455x3.tif]

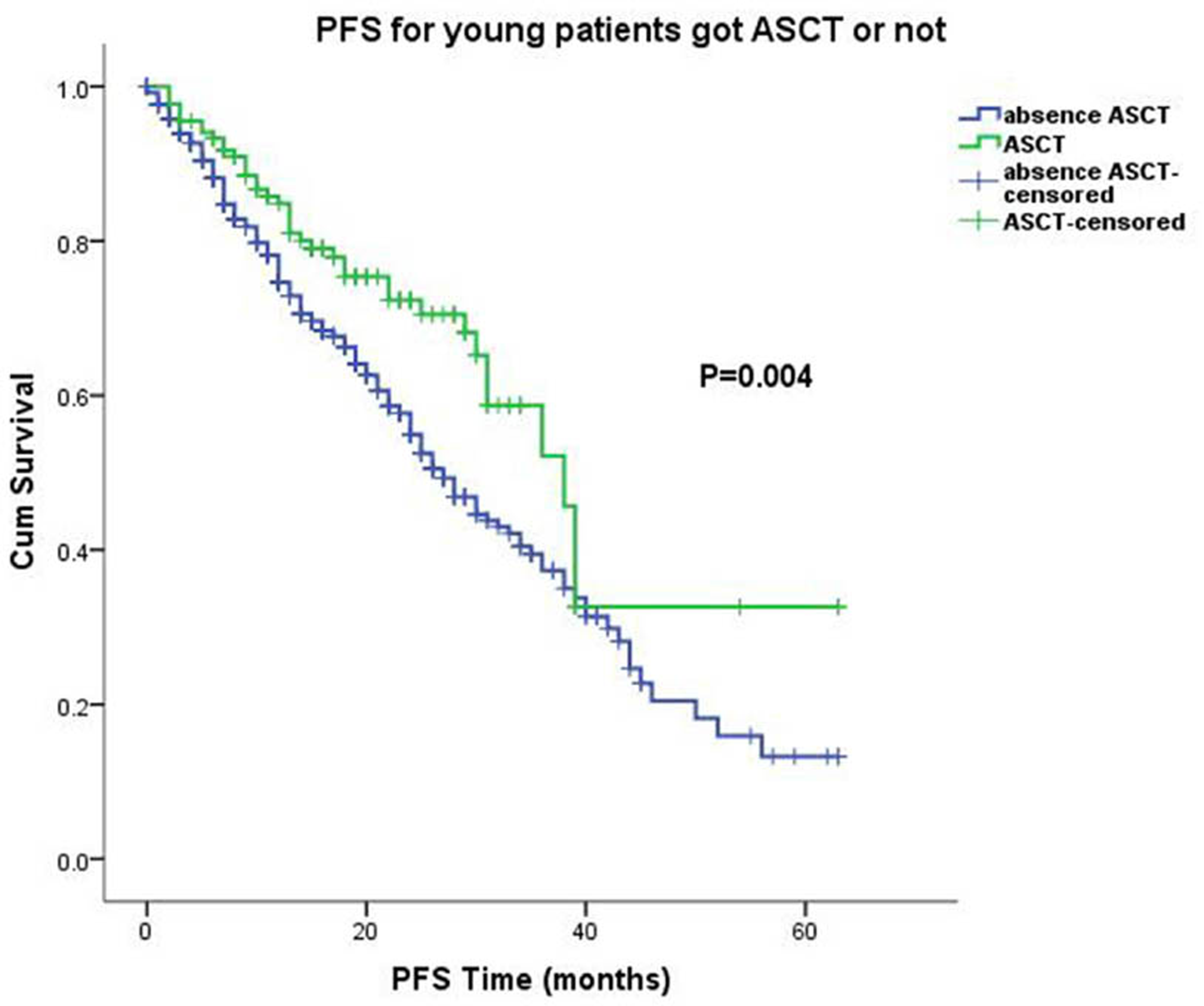

Supplement: Supplementary Figure 4 [file bcj201455x4.tif]

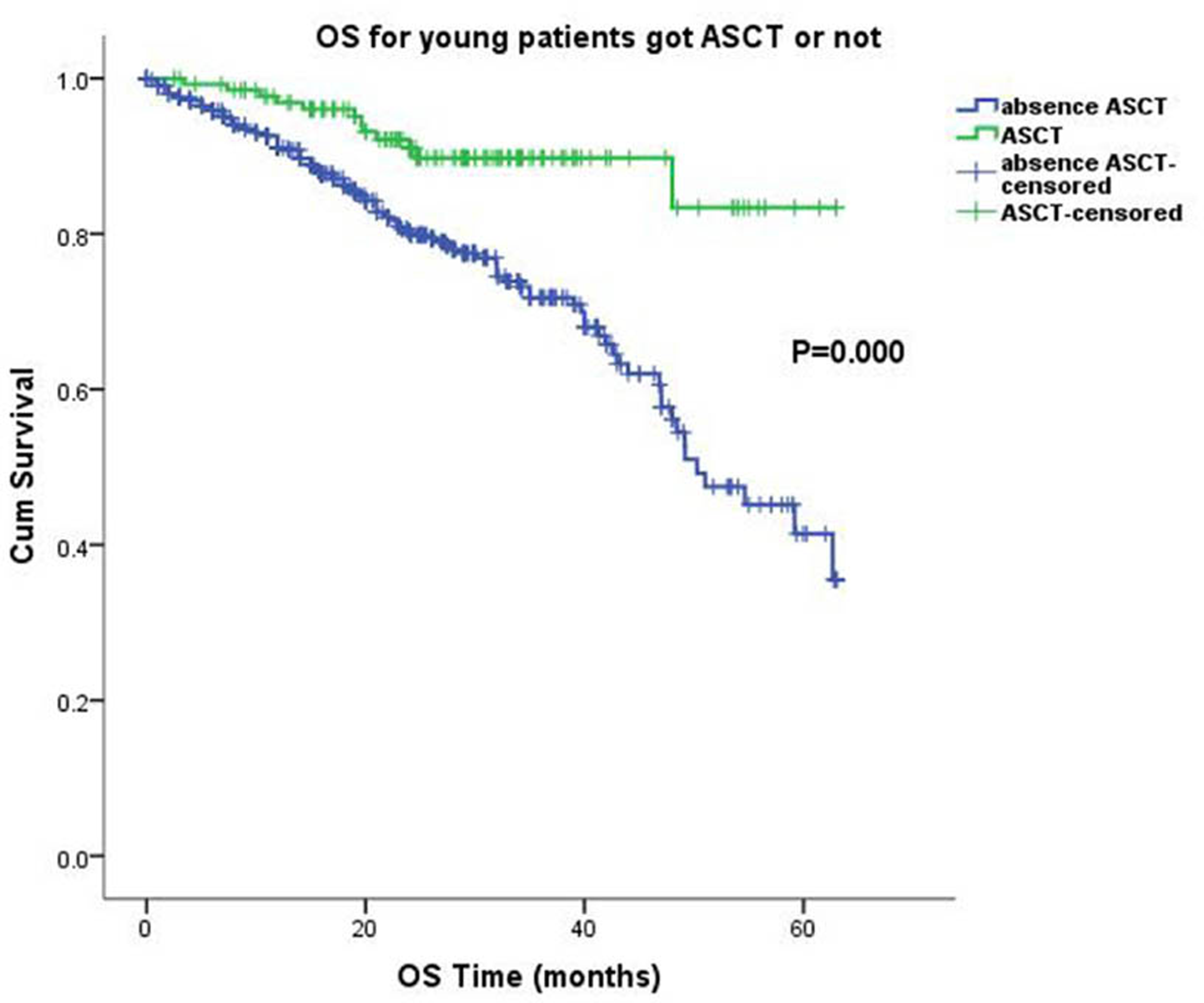

Supplement: Supplementary Figure 5 [file bcj201455x5.tif]

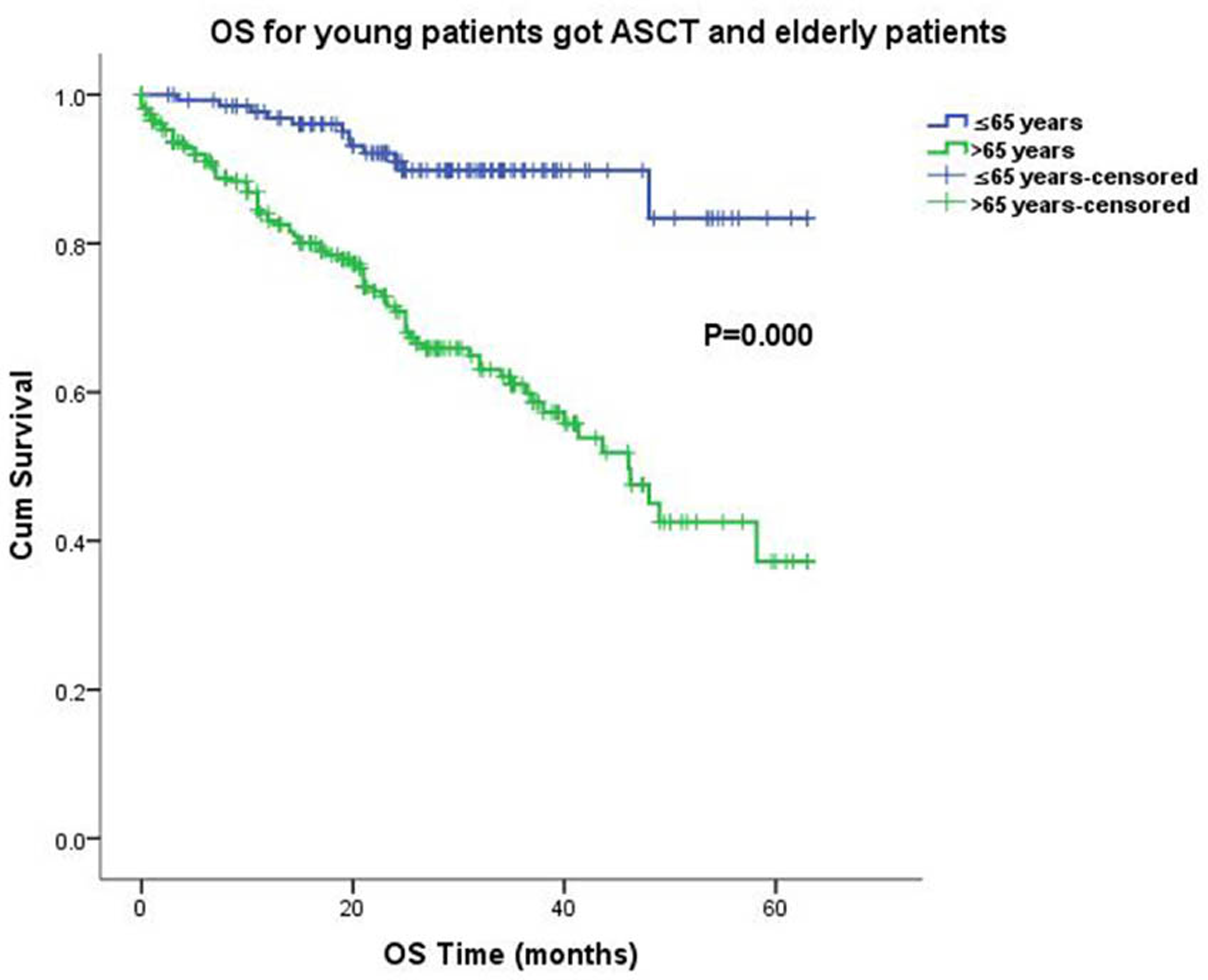

Supplement: Supplementary Figure 6 [file bcj201455x6.tif]

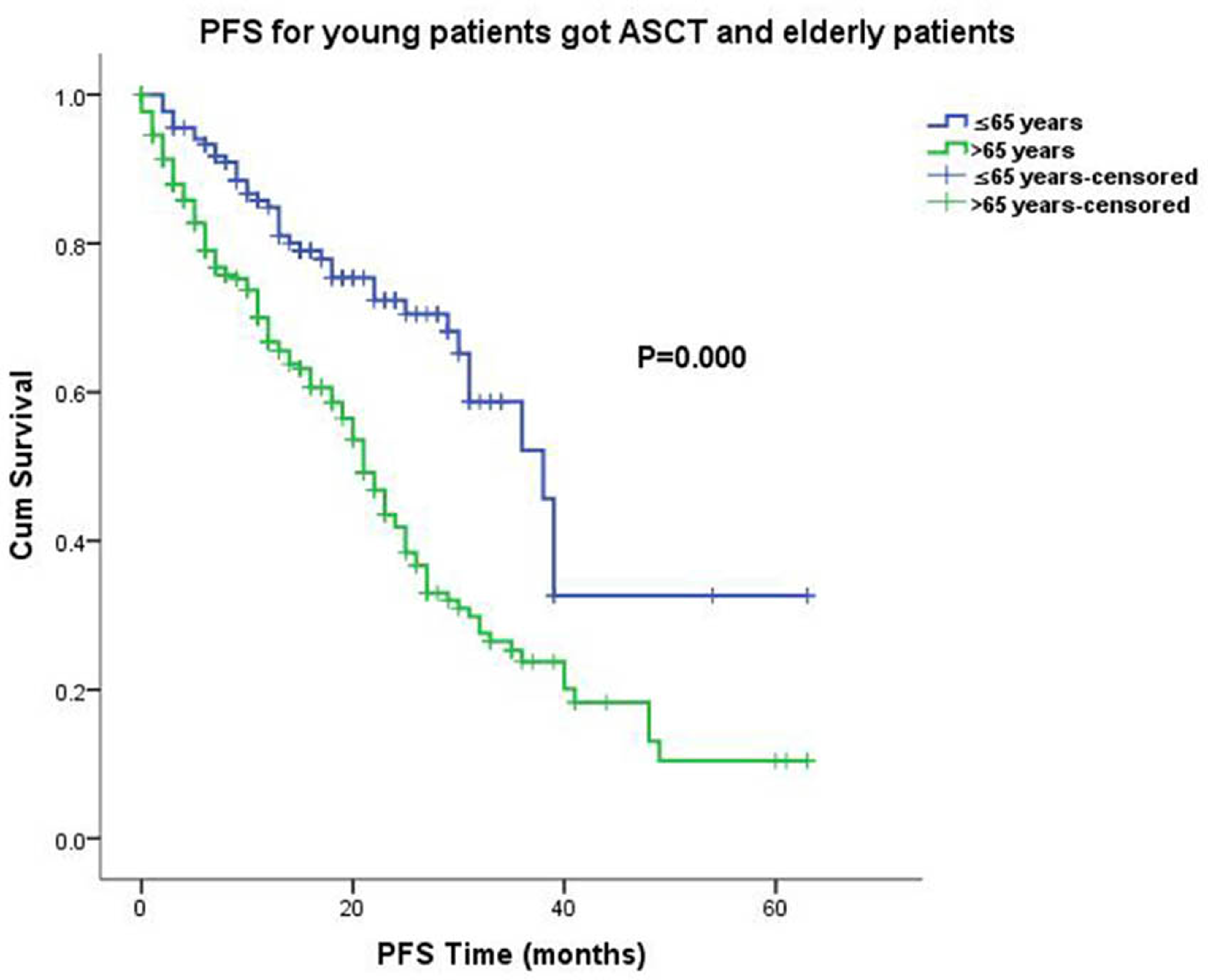

Supplement: Supplementary Figure 7 [file bcj201455x7.tif]

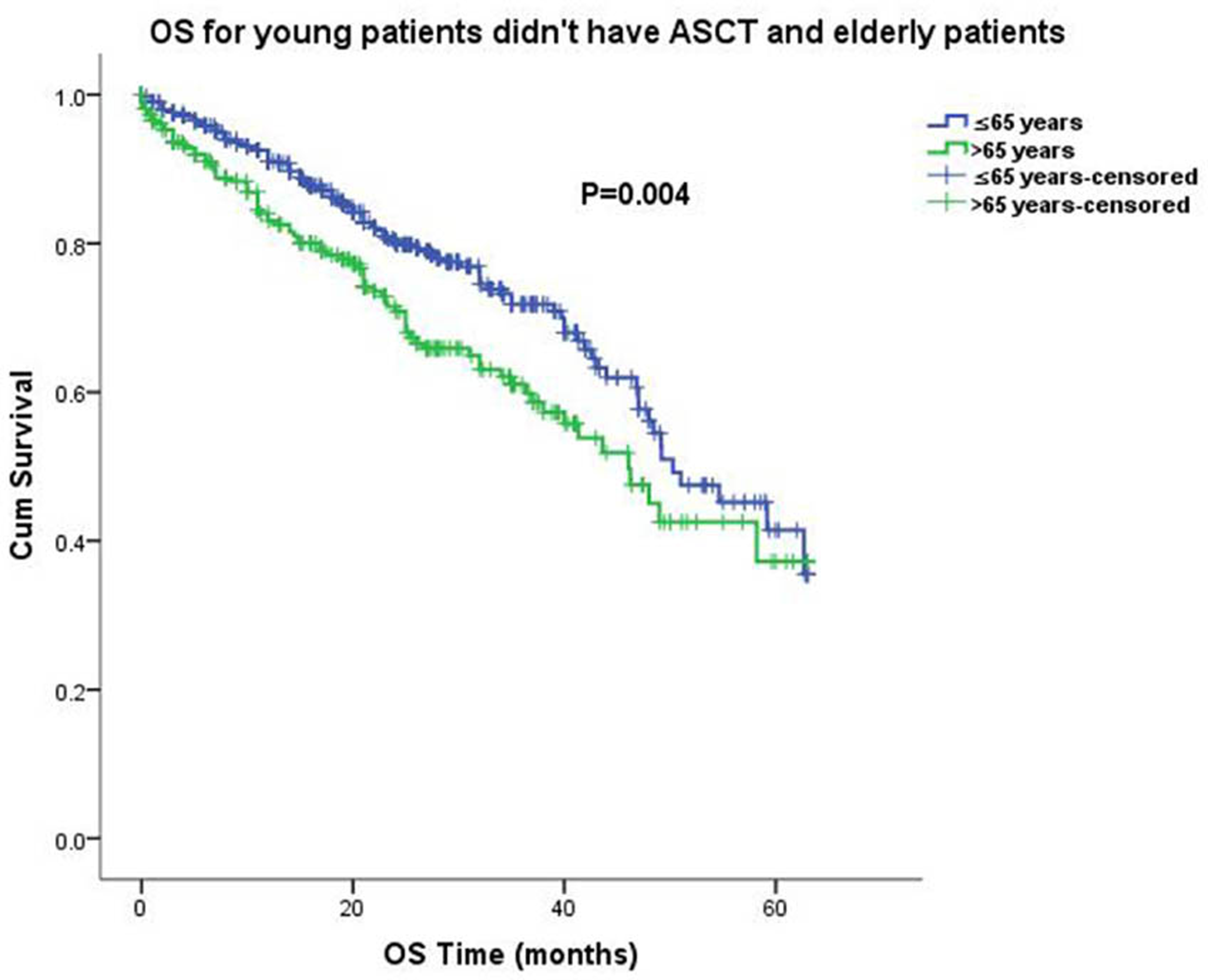

Supplement: Supplementary Figure 8 [file bcj201455x8.tif]

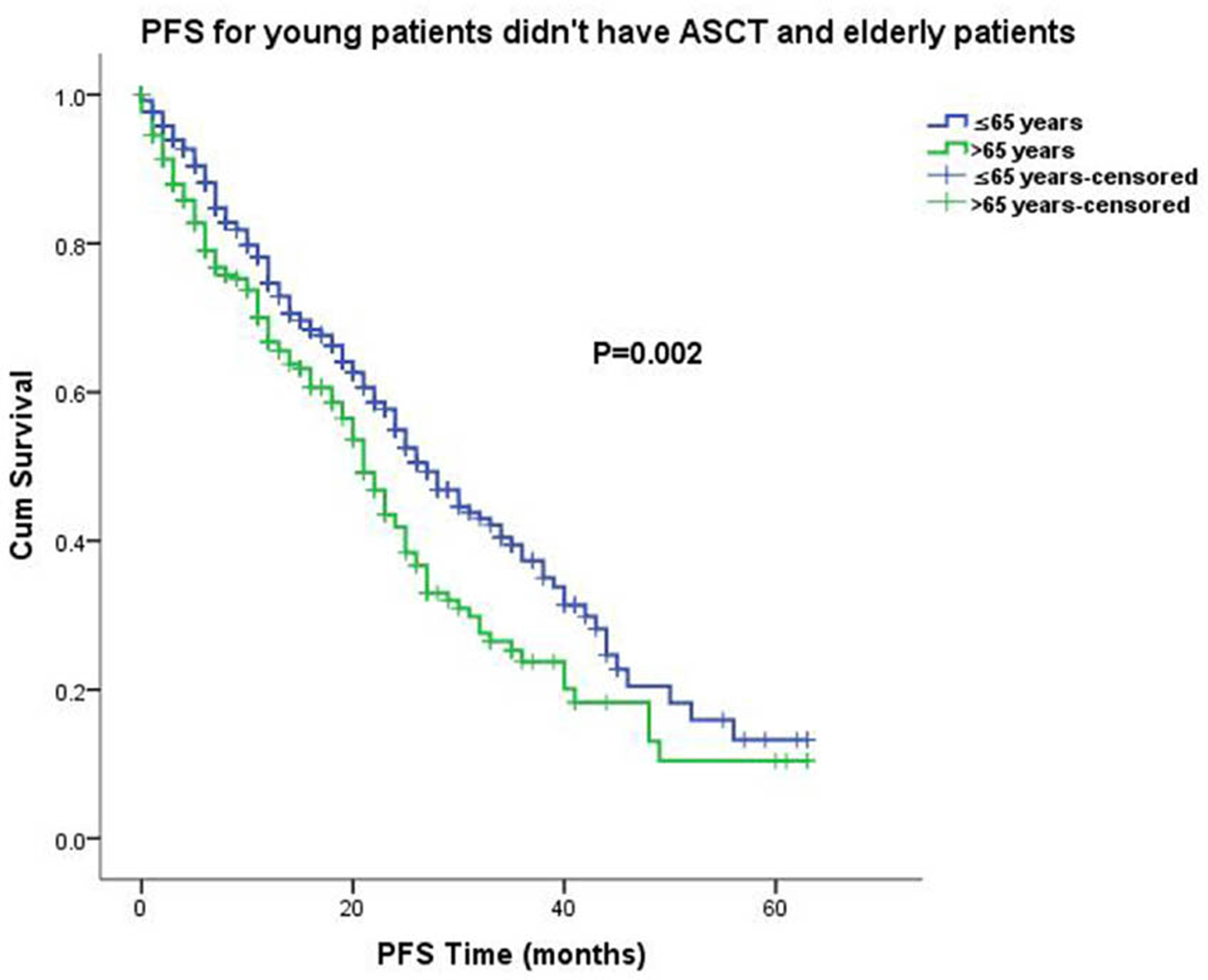

Supplement: Supplementary Figure 9 [file bcj201455x9.tif]
